# Integrated Transcriptome Profiling and Pan-Cancer Analyses Reveal Oncogenic Networks and Tumor-Immune Modulatory Roles for FABP7 in Brain Cancers

**DOI:** 10.3390/ijms252212231

**Published:** 2024-11-14

**Authors:** Yool Lee, Carlos C. Flores, Micah Lefton, Sukanya Bhoumik, Yuji Owada, Jason R. Gerstner

**Affiliations:** 1 Department of Translational Medicine and Physiology, Elson S. Floyd College of Medicine, Washington State University, Spokane, WA 99202, USA; carlos.c.flores@wsu.edu (C.C.F.); micah.lefton@wsu.edu (M.L.); sukanya.bhoumik@wsu.edu (S.B.); 2 Department of Integrative Physiology and Neuroscience, College of Veterinary Medicine, Washington State University, Pullman, WA 99164, USA; 3 Sleep and Performance Research Center, Washington State University, Spokane, WA 99202, USA; 4 Steve Gleason Institute for Neuroscience, Washington State University, Spokane, WA 99202, USA; 5 Department of Organ Anatomy, Graduate School of Medicine, Tohoku University, Seiryo-cho 2-1, Aobaku, Sendai 980-8575, Japan; owada@med.tohoku.ac.jp

**Keywords:** FABP7, NSC, LGG, GBM, TCGA, TIME, Treg, CAF, MDSC

## Abstract

Fatty acid binding protein 7 (FABP7) is a multifunctional chaperone involved in lipid metabolism and signaling. It is primarily expressed in astrocytes and neural stem cells (NSCs), as well as their derived malignant glioma cells within the central nervous system. Despite growing evidence for FABP7’s tumor-intrinsic onco-metabolic functions, its mechanistic role in regulating the brain tumor immune microenvironment (TIME) and its impact on prognosis at the molecular level remain incompletely understood. Utilizing combined transcriptome profiling and pan-cancer analysis approaches, we report that FABP7 mediates the expression of multiple onco-immune drivers, collectively impacting tumor immunity and clinical outcomes across brain cancer subtypes. An analysis of a single-cell expression atlas revealed that FABP7 is predominantly expressed in the glial lineage and malignant cell populations in gliomas, with nuclear localization in their parental NSCs. Pathway and gene enrichment analysis of RNA sequencing data from wild-type (WT) and Fabp7-knockout (KO) mouse brains, alongside control (CTL) and FABP7-overexpressing (FABP7 OV) human astrocytes, revealed a more pronounced effect of FABP7 levels on multiple cancer-associated pathways. Notably, genes linked to brain cancer progression and tumor immunity (ENO1, MUC1, COL5A1, and IL11) were significantly downregulated (>2-fold) in KO brain tissue but were upregulated in FABP7 OV astrocytes. Furthermore, an analysis of data from The Cancer Genome Atlas (TCGA) showed robust correlations between the expression of these factors, as well as FABP7, and established glioma oncogenes (EGFR, BRAF, NF1, PDGFRA, IDH1), with stronger associations seen in low-grade glioma (LGG) than in glioblastoma (GBM). TIME profiling also revealed that the expression of FABP7 and the genes that it modulates was significantly associated with prognosis and survival, particularly in LGG patients, by influencing the infiltration of immunosuppressive cell populations within tumors. Overall, our findings suggest that FABP7 acts as an intracellular regulator of pro-tumor immunomodulatory genes, exerting a synergistic effect on the TIME and clinical outcomes in brain cancer subtypes.

## 1. Introduction

Gliomas are a group of primary brain tumors originating from glial cells in the central nervous system (CNS) [1]. In recent years, the World Health Organization (WHO) has classified gliomas into grades I to IV [2,3], with the 2021 classification emphasizing genetic differences by distinguishing between grade IV astrocytoma and isocitrate-dehydrogenase (IDH)-wildtype glioblastoma [3]. In The Cancer Genome Atlas (TCGA) database, grade II and III tumors are lower-grade gliomas (LGGs), while grade IV tumors are classified as glioblastomas (GBMs). Despite significant advances in surgery, chemotherapy, and radiotherapy, glioma prognosis remains poor, with GBM patients having a median survival of 1.25 years compared to 6.5–8 years for LGG [4,5,6]. Thus, understanding the biological mechanisms affecting glioma survival is crucial for improving diagnosis and treatment strategies.

Fatty acid binding protein 7 (FABP7) is a brain-enriched protein highly expressed in glial cells and neural stem/progenitor cells (NSCs/N.Ps) throughout the nervous system [7,8]. As a lipid chaperone, it regulates the uptake, transport, metabolism, and storage of fatty acids [7,9], influencing functions, such as gene expression, growth, and inflammation [8,10]. The upregulation of FABP7 in various cancers, including brain tumors, has been associated with tumor progression [11,12,13]. At the molecular level, FABP7 promotes FA uptake and lipid droplet (LD) formation [14,15], and it modulates several oncogenic signaling pathways, including those regulated by proto-oncogene tyrosine-protein kinase Src [16], mitogen-activated protein kinase kinase/extracellular signal-regulated kinase (MEK/ERK) [17,18], nuclear factor κB (NF-κB) [10], and Wnt/β-catenin [19,20]. Notably, the nuclear localization of FABP7 has been linked to glioma progression, particularly in oncogene-mutated aggressive subtypes, such as EGFR-amplified [21,22] and IDH1 wild-type GBM [23,24]. The nuclear FABP7 also regulates genes associated with tumor stemness and metastasis in GBM model studies [25], underscoring its transactivational role in brain tumor progression.

Accumulating evidence suggests that the tumor immune microenvironment (TIME), which is composed of a diverse array anti-tumor immune cells, including cytotoxic T lymphocytes (CD8+ T cells), helper T lymphocytes (CD4+ T cells), and immunosuppressive cells, such as regulatory T cells (Tregs), cancer-associated fibroblasts (CAFs), and myeloid-derived suppressor cells (MDSCs) [26], contributes to tumor initiation, progression, and response to therapy [27]. Gene expression levels of oncogenic factors have been shown to play pivotal roles in influencing tumor progression and malignancy by shaping the TIME [26,28]. However, despite the known role of FABP7 in tumor growth and progression, its impact on the TIME and prognosis in LGG and GBM remains largely unknown.

In this study, we performed a comprehensive analysis of large-scale transcriptomic datasets to investigate the oncogenic and immunomodulatory roles of FABP7 in brain cancers, focusing on LGG and GBM. Single-cell expression atlas analysis revealed that FABP7 is predominantly expressed in glial and malignant cell populations within gliomas and shows nuclear localization in NSCs. Our combined comparative gene expression and pan-cancer analysis revealed that FABP7 levels remodel cancer-associated pathways and identify oncogenic and tumor-immune modulatory factors that enhance immunosuppressive infiltrates, significantly impacting clinical outcomes in brain cancers, particularly LGG. These findings highlight the significant role of FABP7 in shaping pro-tumor microenvironments, providing valuable insights into brain cancer pathogenesis and treatment mechanisms.

## 2. Results

### 2.1. FABP7 Is Predominantly Expressed in Normal and Malignant Glial Cell Populations, Exhibiting Nuclear Localization in Their Parental NSCs

FABP7 has been suggested to play an oncogenic role in various tumors [11,12,13]. To investigate the relative expression profiles of FABP7 in different cancer types, we utilized the Human Protein Atlas (HPA) database and found that FABP7 exhibits higher levels of expression in multiple cancer tissues compared to normal tissues, with the highest levels observed in glioma, followed by renal cancer, head and neck cancer, breast cancer, and skin cancer (Figure S1A). 

In parallel, the immunohistochemical staining data revealed that the expression levels of FABP7 protein in tumor tissues were significantly higher than in normal tissues, particularly in gliomas, with gradual declines in staining intensity in other tumor types (Figure S1A). To further examine *FABP7* mRNA expression profiles in gliomas at the single-cell level, we analyzed public, single-cell RNA-seq (scRNA-seq) datasets and found that *FABP7* is most highly expressed in malignant glioma cell populations, with notably comparable expression in normal astrocytes and oligodendrocyte precursor cells (OPCs), a type of non-neuronal glial cell, among other cell types (Figures S1B and S2). In contrast, *FABP7* expression was not detected in any tumor-associated immune cells, such as CD8+ T cells, CD4+ T cells, and Tregs (Figures S1C and S2). These results suggest that FABP7 in glial cell lineages may play a major carcinogenic role in brain tumor development and progression.

In addition to the primary tumor tissue analysis data, we examined the expression of FABP7 in multiple cell lines that exhibit phenotypic resemblance to various organs or tumor tissues (Figure S3). Interestingly, we observed that *FABP7* mRNA expression was conspicuously expressed only in the AF22 neuroepithelial stem (NES) cell line, which is derived from human induced pluripotent stem cells (iPSCs) [29,30], and the U-251 MG glioblastoma cell line, generated from a grade III-IV malignant astrocytoma from a 75-year-old male that expresses EGFR and contains GFAP-positive cells [31,32,33] (Figure S3A). Furthermore, an analysis of immunostaining data revealed that FABP7 expression is exclusively localized in AF22 cell nuclei, while in U-251 MG cells, it is evenly distributed across the nucleus and cytoplasm, with higher intensity (Figure S3B). Recent studies suggest that neural stem cells (NSCs), undifferentiated cells with self-renewal and multipotent capacities, are the potential origin of GBM, as they can mutate into glioma stem cells (GSCs) linked to tumor recurrence and therapy resistance or differentiate into neurons, astrocytes, and OPCs, the latter two of which can become malignant [34]. This suggests that the differing intracellular properties of NSCs and their differentiated glioma cells could influence FABP7 localization and function.

### 2.2. FABP7 Levels Remodel the Transcriptional Landscape of Cancer-Associated Pathways in Mouse Brain Tissue and Human Astrocytes

It has recently been shown that FABP7 translocates to the nucleus and interacts with nuclear factors (e.g., ACLY, RXRα) to modulate gene expression associated with cellular physiology and metabolism in both astrocytes and glioma cells [23,24,25]. To investigate how the FABP7 function influences global gene expression profiles in the brain, we performed RNA-seq analysis on cortical samples from *Fabp7*-KO (*n* = 4) and wild-type (WT) control mice (*n* = 5; see Section 4). A heatmap analysis of the resulting transcriptomic data showed comprehensive modifications in gene expression, with numerous genes being either downregulated or upregulated in *Fabp7*-KO brain samples compared to WT samples (WT vs. *Fabp7*-KO), indicating the regulatory role of FABP7 in modulating gene expression in the brain (Figure S4A). To determine the potential impact of FABP7 null on physio-pathological pathways, we conducted integrated and comparative Kyoto Encyclopedia of Genes and Genomes (KEGG) pathway enrichment analyses using the transcriptome datasets. The KEGG analyses, applied to all detected genes that were up- or downregulated in *Fabp7*-KO mouse cortical tissues relative to WT tissues (WT vs. *Fabp7*-KO), unveiled various biological and disease pathways with significant differential gene enrichment (DGEn) profiles (meeting the 0.05 false discovery rate (FDR) criterion). Interestingly, notable DGEn was observed for eight terms linked to cancer out of the top 20 terms (lowest FDRs) identified, including pathways in cancer, hepatocellular carcinoma (HCC), microRNAs in cancer, and proteoglycans in cancer, as well as terms for the Hippo, ras-proximate-1 (RAP1), mitogen-activated protein kinase (MAPK), and phosphoinositide 3-kinase (PI3K)-AKT signaling pathways (Figure S4B,C). 

**Figure 1:**
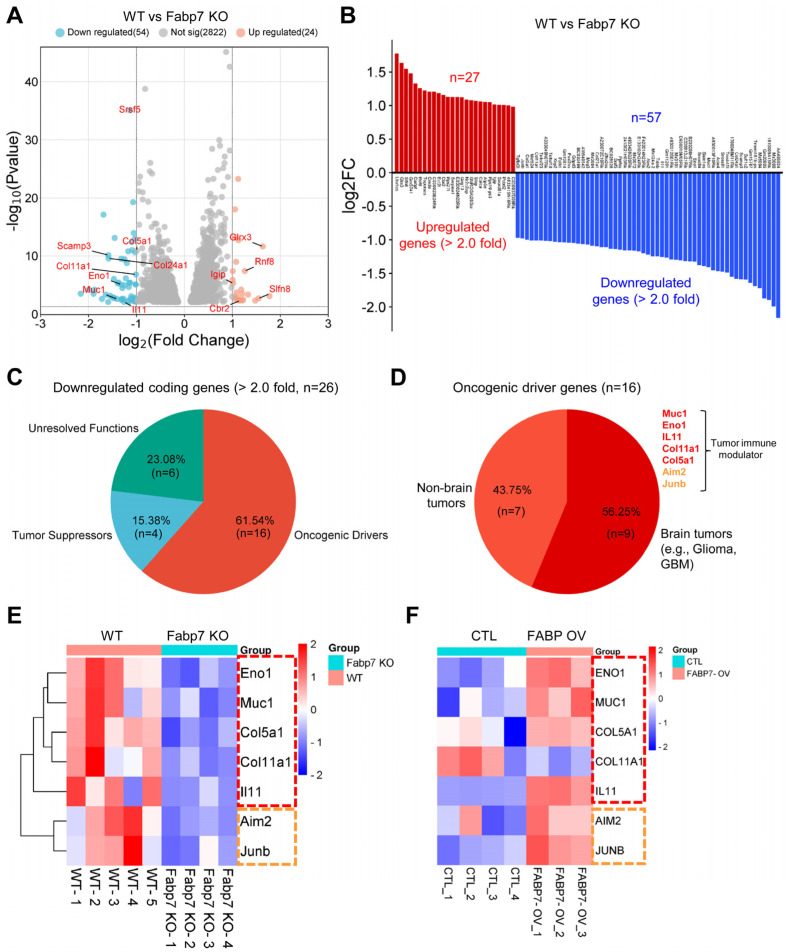
FABP7 modulates the expression of genes associated with brain cancer progression and tumor immunity. (**A**) Volcano plot showing differential gene expression in cortical tissues from wild-type (WT) and Fabp7-knockout (KO) mice (adjusted *p*-value ≤ 0.05, fold change cutoff: 2.0). (**B**) Log2 fold-change values for significantly upregulated (red) and downregulated (blue) genes in Fabp7-KO compared to WT tissues. (**C**) Pie chart illustrating the characteristics and functions of downregulated coding genes (≥2-fold change, *n* = 26) categorized into the following: Oncogenic Drivers (*n* = 16, red), Tumor Suppressors (*n* = 4, bright blue), and Unresolved Functions (*n* = 6, blue-green) (see Dataset S1 for details). (**D**) Oncogenic driver genes identified in (**C**) further categorized into those functional in non-brain tumors (*n* = 7, orange) and brain tumors (*n* = 9, red), with immunomodulatory functions noted. (**E**) Heatmap of tumor immunomodulatory gene expression (TIMGs) in WT (*n* = 5) vs. Fabp7-KO (*n* = 4) samples, with brain and non-brain TIMGs marked by dashed red and orange lines, respectively. (**F**) Heatmap of TIMG expression in control (CTL, *n* = 4) and FABP7-overexpressing (FABP7 OV, *n* = 3) human astrocytes, with markings as in (**E**).

To delve into the differential gene expression (DGEx) profiles of cortical WT and *Fabp7*-KO mouse tissues, we performed volcano plot analysis of all the significantly up- or downregulated genes (adjusted *p*-value [padj] < 0.05) that showed more than a 2-fold change in expression. Our results showed a higher number of significantly downregulated genes (*n* = 54) than upregulated genes (*n* = 24) in *Fabp7*-KO cells compared to WT cells (Figure 1A,B), which aligns with our hypothesis that FABP7 has a nuclear role in gene activation [25]. 

Literature-based annotations of the protein-coding genes (*n* = 26) downregulated in *Fabp7*-KO cells indicated that over half of these genes (*n* = 16, 61.54%) are oncogenic drivers that promote tumor progression by playing roles in proliferation, survival, metastasis, and drug resistance (Figure 1C, Dataset S1). The remaining downregulated genes included tumor suppressors (*n* = 4, 15.38%) that counteract cancer development and genes with unresolved functions (*n* = 6, 23.08%) (Figure 1C, Dataset S1). Further annotations revealed that among the oncogenic drivers (*n* = 16), nine genes (56.25%) have exhibited oncogenic functions in brain tumors (e.g., glioma, GBM) and seven genes (43.75%) have such functions in non-brain tumors. Notably, five of the brain onco-driver genes (enolase 1 (*Eno1*), mucin 1 (*Muc1*), collagen type V alpha 1 chain [*Col5a1*], collagen type V alpha 1 chain (*Col11a1*), and interleukin 11 (*Il11*)) (Table 1), and two of the non-brain onco-driver genes (absent in melanoma 2 (*Aim2*) and Jun B proto-oncogene (*Junb*)) have been reported to regulate tumor immunosurveillance and therapy responses [35,36] (Figure 1D, Dataset S1).

A heatmap analysis further clarified the marked downregulation of these tumor immunomodulatory genes (TIMGs) in *Fabp7*-KO brain tissues compared to WT brain tissues (Figure 1E). These results indicate that FABP7 plays a role in modulating pro-tumoral gene expression in the brain.

To further explore FABP7’s impact on global gene expression, we analyzed the transcriptomic changes that occurred in cultured human astrocytes (derived from induced pluripotent stem cells (iPSCs) of healthy control subjects) that were engineered to overexpress FABP7 [10]. GO and KEGG pathway analyses of all the genes that were either significantly up- or downregulated (adjusted *p*-value [padj] < 0.05) in FABP7-overexpressing (FABP7 OV) astrocytes compared to control (CTL) astrocytes unveiled a DGEn profile that was predominantly composed of 10 cancer-associated terms, including Pancreatic cancer, Colorectal cancer, Choline metabolism in cancer, MicroRNAs in cancer, and Proteoglycans in cancer, as well as terms related to Hippo, RAP1, MAPK, and PI3K–AKT signaling, mirroring the results of the pathway analysis performed on the genes that were significantly altered in cortical *Fabp7*-KO tissues (Figures S4A,B and S5A,B). A heatmap analysis of the differentially expressed genes showed that most of the genes associated with signaling pathways in cancer were more highly expressed in FABP7 OV astrocytes than in CTL astrocytes (Figure S5C). Moreover, as anticipated, most of the TIMGs that were downregulated in *Fabp7*-KO cells (all except for *COL11A1*) were extensively upregulated in FABP7 OV astrocytes compared to CTL cells (Figure 1F). These results underscore a potential role for FABP7 in shifting transcriptional landscapes in brain cells to create pro-tumoral intracellular environments.

### 2.3. FABP7 Exhibits Stronger Correlations with Oncogenic and Tumor Immunomodulatory Factors in LGG than in GBM

**Figure 2:**
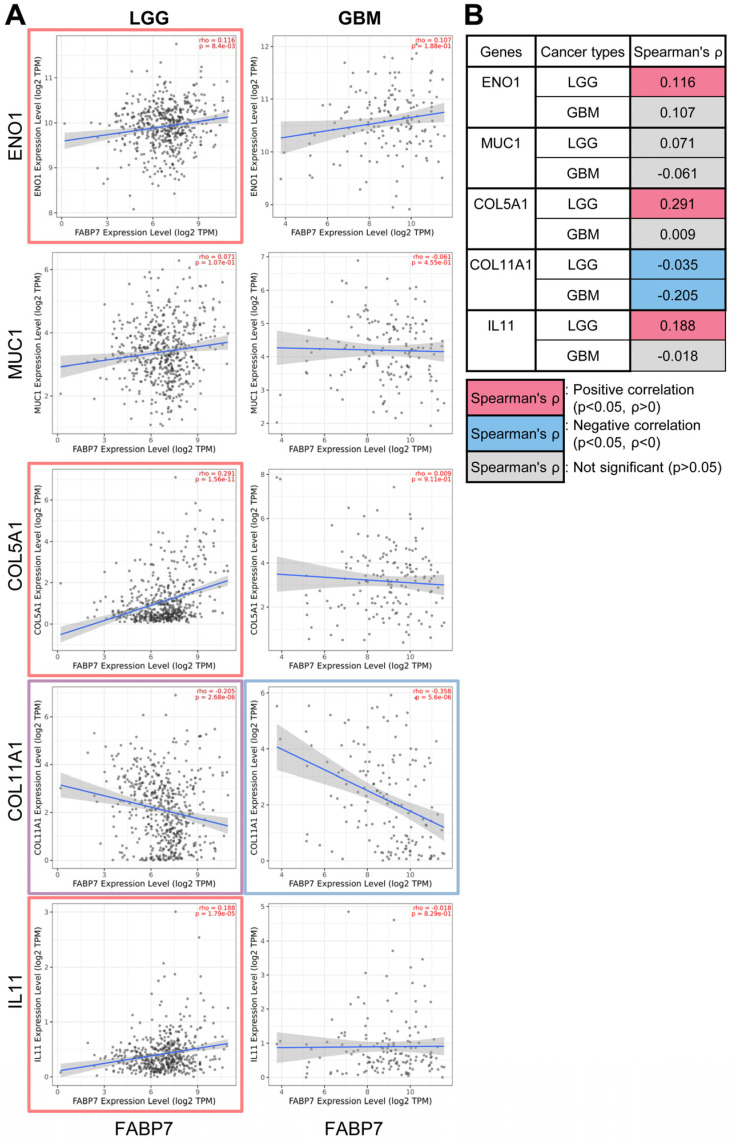
*FABP7* expression is more strongly correlated with the expression of its modulated factors in LGG than GBM. (**A**) Correlations between *FABP7* expression and the expression levels of its regulated brain tumor immunomodulatory genes (identified in Figure 1C–F) were assessed in low-grade glioma (LGG, *n* = 516) and glioblastoma (GBM, *n* = 153) using the Gene_Corr module in the Tumor Immune Estimation Resource (TIMER) database (see Section 4 for details). Genes with significantly positive or negative correlations (*p* < 0.05), based on purity-adjusted partial Spearman’s rho values, are indicated with red and blue boxes, respectively. (**B**) Table of the purity-adjusted partial Spearman’s rho values indicating the degree of correlation between *FABP7* expression and the expression of the brain tumor immunomodulatory genes that it regulates, as shown in (**A**). Abbreviations: ENO1, Enolase 1; MUC1, Mucin 1; COL5A1, Collagen Type V Alpha 1 Chain; COL11A1, Collagen Type XI Alpha 1 Chain; IL11, Interleukin 11.

Based on our transcriptomic findings, we extended our investigation to probe the role of FABP7 and its regulated genes (as identified in Figure 1 in human patient tumors). For these studies, we examined the differential expression of *FABP7* and its modulated genes in tumor and adjacent normal tissues across all tumors in The Cancer Genome Atlas (TCGA). Consistent with the results from non-tumor samples, all the TIMGs that we identified (*ENO1*, *MUC1*, *COL5A1*, *COL11A1*, *IL11*, *AIM2*, *JUNB*) as well as *FABP7*, exhibited significantly or moderately elevated expression in multiple TCGA tumors, including GBM, compared to normal tissues (Figure S6). A specific analysis of brain cancer types using Spearman’s correlation coefficient (*ρ*) revealed that *FABP7* expression exhibited strong correlations (*p* < 0.05, *ρ* > 0) with the expression of most of its modulated factors in LGG (significantly positive correlations with *ENO1*, *COL5A1*, and *IL11*, and a negative correlation with COL11A1), in contrast to GBM in which little correlation was observed (Figure 2A,B). 

**Figure 3:**
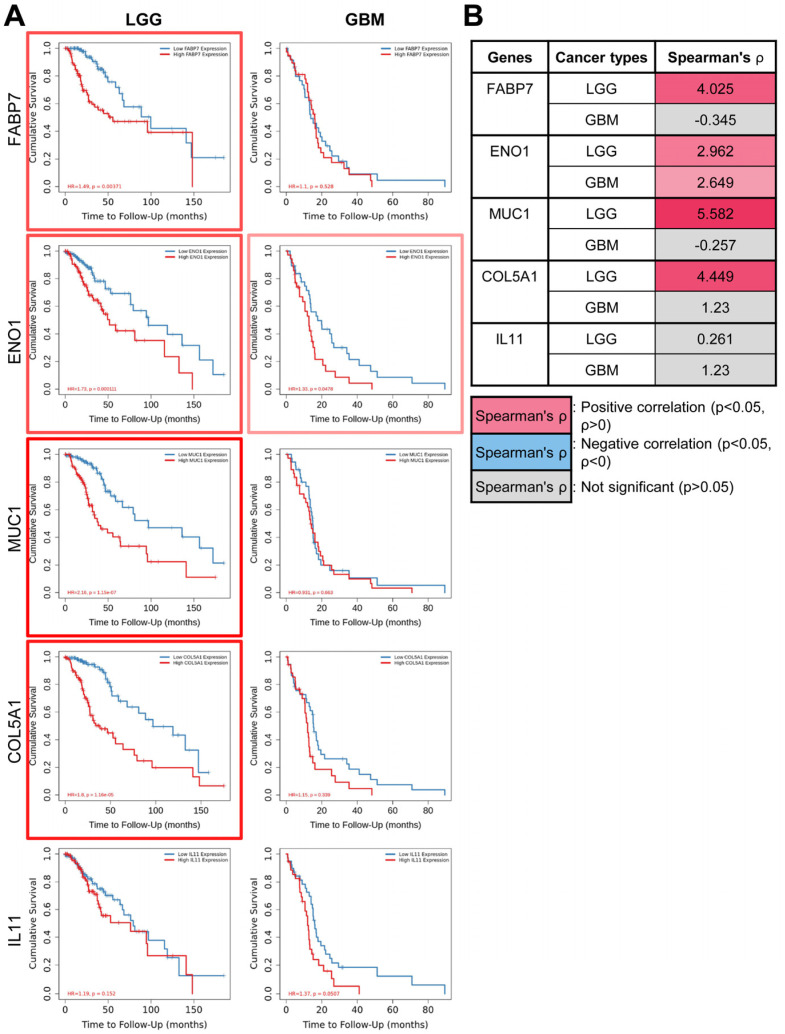
The expression of *FABP7* and its modulated genes is more highly correlated with patient prognosis and outcomes in LGG than GBM. (**A**) Survival curves for brain cancer patients with low-grade glioma (LGG) and glioblastoma (GBM) were generated based on the expression levels of *FABP7* and its modulated genes, as indicated. Data were obtained from the TIMER database and are represented using the Cox proportional hazard model. Kaplan–Meier (KM) curve parameters were applied to evaluate the significance of gene expression outcomes in both LGG and GBM. The hazard ratio and *p*-value for the Cox model, as well as the log-rank *p*-value for the KM curve, are shown on each KM curve plot. Genes with positive correlations (*p* < 0.05), based on purity-adjusted partial Spearman’s rho values, are indicated by boxes of varying shades of red to indicate significance, as shown in (**B**). (**B**) Table of the purity-adjusted partial Spearman’s rho values indicating the degree of correlation between *FABP7* and its regulated factors, as described in (**A**). The analysis was adjusted for clinical factors, such as age, stage, and purity (see Section 4 for details). Abbreviations: FABP7, Fatty Acid Binding Protein 7; ENO1, Enolase 1; MUC1, Mucin 1; COL5A1, Collagen Type V Alpha 1 Chain; IL11, Interleukin 11.

Notably, this correlation pattern aligned with the expression data from FABP7 OV astrocytes in which only *COL11A1* exhibited an inverse expression pattern compared to the other genes (Figure 1F). We also found that the expression of *FABP7* and its co-regulated genes showed similar patterns of significant correlations with patient prognosis and survival. These correlations were particularly notable in LGG compared to GBM (Figure 3A), with positive correlations (*p* < 0.05, *ρ* > 0) being observed for *ENO1*, *MUC1*, and *COL5A1* (Figure 3B). 

The preferential correlation of *FABP7* expression with LGG was further revealed through another survival analysis that measured hazard ratios (HRs). In this analysis, *FABP7* expression displayed a significantly higher correlation with increased cancer risk in LGG but not GBM, compared to other cancer types (Figure S7). In conjunction with these findings, it was also observed that *FABP7* expression is preferentially correlated in LGG over GBM (*p* < 0.05, Log2 fold-change [Log2FC]) with the mutational status of several established oncogenes (*EGFR*, *BRAF*, *NF1*, *PDGFRA*, *IDH1*) for which mutations are tightly associated with the development and progression of brain tumors [47] (Figure S8). Notably, tumors with mutant *IDH1* showed a slightly higher negative correlation (*p* < 0.05, Log2FC < 0) with *FABP7* expression in GBM (Log2FC: −0.472) compared to LGG (Log2FC: −0.314), while the mutational statuses of the other oncogenes examined displayed highly positive correlations (*p* < 0.05, Log2FC > 0) with *FABP7* expression, particularly in LGG (Figure S8B). This aligns with recent studies showing that nuclear FABP7 levels are higher in wild-type IDH1 malignant GBM tumors compared to IDH1-mutant LGG tumors, differentially regulating cell proliferation and migration in these distinct tumor types [23,25]. Overall, these findings suggest that FABP7 may play a significant role in the regulatory network of genes promoting brain tumor development, especially in LGG.

### 2.4. Expression Levels of FABP7 and Its Modulated Onco-Immune Drivers Correlate with Clinical Outcomes in LGG and GBM by Promoting the Tumor Infiltration of Immunosuppressive Cell Types

**Figure 4:**
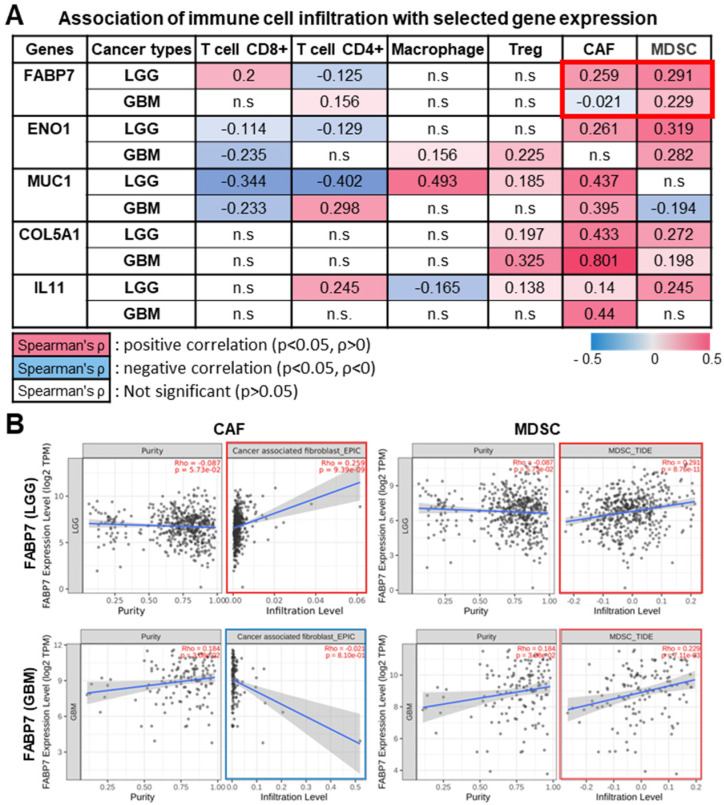
The expression of *FABP7* and its modulated factors impact the tumor-immune microenvironment of LGG and GBM by enhancing immunosuppressive infiltrates. (**A**) Table showing the associations between the expression of the indicated genes (*FABP7*, *ENO1*, *MUC1*, *COL5A1*, *IL11*) and immune cell (CD4+ T cells, CD8+ T cells, macrophages, Tregs, CAFs, and MDSCs) infiltration in low-grade glioma (LGG) and glioblastoma (GBM). (**B**) Representative Kaplan–Meier (KM) curves illustrating significantly positive (boxed in red) and negative (boxed in blue) correlations between the expression of *FABP7* and the infiltration of the indicated immune cells (CAFs and MDSCs) in LGG and GBM are shown. The purity-adjusted Spearman’s rank correlation test was applied to determine both the *p*-values and partial correlation (cor) values. Significantly positive and negative correlations were determined, based on purity-adjusted Spearman’s rho values (*p* < 0.05). Abbreviations: T cell CD8+, cytotoxic t lymphocytes; T cell CD4+, helper T lymphocytes, Treg, regulatory T cells; CAF, cancer-associated fibroblasts; MDSC, myeloid-derived suppressor cells; FABP7, Fatty Acid Binding Protein 7; ENO1, Enolase 1; MUC1, Mucin 1; COL5A1, Collagen Type V Alpha 1 Chain; IL11, Interleukin 11; n.s, non-significant.

In addition to their intracellular functions, oncogenic factors can also impact tumor progression and prognosis by shaping the interactions between tumor cells and immune cells within the tumor microenvironment [26,28]. The tumor microenvironment comprises a heterogeneous population of anti-tumor immune cells (e.g., CD8+ T cells and CD4+ T cells) and pro-tumor suppressive cells (e.g., Tregs, CAFs, and MDSCs) [26]. To understand the role of FABP7 and its modulated genes in tumor immunity, we investigated the association between their expression and the abundance of tumor-infiltrating immune cells (TIICs) in brain cancers, particularly LGG and GBM. Interestingly, we observed an overall significantly positive correlation between the expression of *FABP7* and its modulated genes (*ENO1*, *MUC1*, *COL5A1*, and *IL11*) and the infiltration of immunosuppressive cells (Tregs, CAFs, and MDSCs) relative to anti-tumor immune cells (CD8+ T cells, CD4+ T cells, and macrophages) in LGG compared to GBM (Figure 4A). 

Notably, *FABP7* expression was significantly associated with both CAF and MDSC infiltration in LGG, but only MDSC infiltration in GBM. Intriguingly, CAF infiltration was positively correlated with *FABP7* expression in LGG but was negatively correlated with *FABP7* expression in GBM (Figure 4B). These findings align, in part, with our prior data (Figure 2, Figure 3 and Figure S8) indicating higher and more significant correlations between the expression of *FABP7* and its regulated factors and prognosis and outcomes in LGG patients compared to GBM patients. Collectively, these findings suggest that FABP7, in concert with the factors that it regulates, fosters a pro-tumor immunosuppressive TIME that influences tumor development and patient outcomes in different brain cancer subtypes.

## 3. Discussion

Accumulating studies have shown a pro-tumoral role for FABP7, focusing on intracellular mechanisms within tumor cells and their influence on cell proliferation and migration [11,12]. Despite these advances, it is largely unknown how FABP7 cooperates with other oncogenic drivers at the intracellular level to influence intercellular tumor immunity, particularly in the context of brain cancers. In line with growing evidence indicating a nuclear transactivational function for FABP7 in normal glia and glioma cells, our unbiased analysis of large-scale transcriptomic data—combined with the analysis of LGG and GBM patient tumors and outcomes—revealed that FABP7 regulates multiple onco-immune drivers to foster immunosuppressive environments within tumors, collectively impacting prognosis and survival.

Previous research has focused on FABP7’s role in the cytoplasm, particularly its involvement in fatty acid transport and metabolism, which is crucial during brain development. Indeed, FABP7 is expressed in radial glia or neural progenitor cells during development via a process driven by the activation of Notch receptors [48,49]. FABP7 has also been widely associated with synaptic plasticity, memory, sleep, circadian rhythms, and various brain injuries/diseases through mechanisms, such as the activity-dependent polyadenylation of its mRNA, local translation, circadian clock-controlled expression, and rhythmic trafficking of its mRNA (recently reviewed in [8]). Research on cancer has similarly documented FABP7’s cytoplasmic and metabolic functions, such as modulating fatty acid uptake and processing, lipid droplet formation, and oncogenic signaling pathways [11,12]. However, growing evidence from brain cancer model studies has suggested a novel nuclear role for FABP7. For example, EGFR activation, a frequent feature in GBM, primarily through genomic amplification, has been shown to upregulate FABP7 expression and induce its nuclear translocation in vitro [21]. Furthermore, FABP7 was recently found to interact with nuclear factors, such as ACLY and RXRα, to regulate gene expression in astrocytes and glioma cells, influencing cellular metabolism and proliferation [23,24,25]. Our study supports this emerging understanding of the role of nuclear FABP7 in brain tumor development and progression. Indeed, we observed FABP7 expression primarily in the nuclei of AF22 cells (compared with U-251MG), a well-established model for neural stem cells (NSCs), which serve as progenitor cells for both glia and malignant GBM cells (Figure S3B) [29,30,34]. However, it is important to note that our findings are based on only two brain-derived tumor cell lines, AF22 and U-251MG. While these cell lines offer valuable insights into FABP7 expression, they may not fully represent the heterogeneity seen across different types and grades of brain tumors. Expanding this research to include a broader range of cell lines, as well as primary tumor samples, would improve the generalizability of these findings and provide a more nuanced understanding of FABP7’s role across various tumor contexts.

Notably, multiple onco-driver genes identified in our transcriptomic analyses showed inverse expression patterns when FABP7 function was lost or gained (Figure 1E,F). In addition, FABP7 overexpression in astrocytes led to the upregulation of most cancer-associated genes, suggesting a potential role for FABP7 in shaping the oncogenic gene expression landscape (Figure S5C). We also found that the expression of the onco-immune factors (*ENO1*, *MUC1*, *COL5A1*, *IL11*) that we identified was significantly correlated with *FABP7* expression in patient brain cancers, particularly LGG (Figure 2), suggesting the pathological relevance of the results of our transcriptomic analyses. 

High levels of FABP7 expression have been linked to poor prognosis in various cancers, including malignant gliomas [11,12,50]. However, our analysis of the available TCGA gene expression profiles showed that the expression of *FABP7*, as well as its co-regulated factors, exhibited stronger correlations with prognosis and the mutational statuses of other established oncogenes in LGG relative to GBM (Figure 3, Figure 4, Figures S7 and S8). Related to our findings, a recent study of the TCGA-GBM transcriptomic dataset reported that low *FABP7* expression is significantly correlated with a shorter overall survival time in IDH-wildtype malignant GBM patients [51]. In addition, a data-independent acquisition mass spectrometry (DIA-MS)-based proteomics analysis of 50 tumor and 30 serum samples from short- and long-term survivors (STS and LTS, respectively) of IDH-wildtype GBM found that FABP7 levels were lower in tumors of the STS group than in tumors of the LTS group [51]. In line with these results, high FABP7 levels have been associated with better responses to chemotherapy in estrogen-receptor-negative breast cancer patients, leading to longer recurrence-free survival [52]. These seemingly contradictory findings are thought to be attributed in part to FABP7’s ligand-dependent activity. FABP7 binds preferentially to polyunsaturated fatty acids, with a stronger affinity for docosahexaenoic acid (DHA) than arachidonic acid (AA). It has been suggested that an AA-rich environment promotes FABP7-expressing GBM cell growth, while a DHA-rich environment inhibits tumor infiltration [53,54]. The precise mechanisms of FABP7’s effects on chemosensitivity and prognosis are unclear, but they may involve increased DHA uptake facilitated by FABP7 in malignant tumor cells, as was reported for GBM NSCs [53]. Supporting this assumption, several studies have indicated that DHA not only stimulates apoptosis but also increases cancer cell responsiveness to chemotherapy by generating reactive oxygen species (ROS) [55,56,57]. Therefore, it is speculated that lower levels of FABP7 in GBM tissues could contribute to worse prognosis through reduced DHA uptake and lower cytotoxic ROS levels [51]. In conjunction with our data, this evidence suggests that high FABP7 expression levels, particularly in primary GBM tissues, may not consistently result in more favorable intra- and extracellular environments for malignant tumor cell survival and maintenance nor lead to a worse prognosis compared to tumors with lower FABP7 expression levels.

Notably, our TIME analysis showed that the expression of *FABP7* and its modulated genes tends to correlate more strongly with abundant immunosuppressive cell populations (e.g., Tregs, CAFs, MDSCs) in LGG compared to GBM (Figure 4). Specifically, *FABP7* expression in LGG positively correlates with both CAF and MDSC infiltration, whereas in GBM, it positively correlates with MDSC infiltration and correlates negatively with CAF infiltration (Figure 4). Recently, a growing number of pan-cancer spatially resolved single-cell analyses have revealed that distinct functional subgroups of CAFs play a pivotal role in tumor angiogenesis and shaping the immunosuppressive microenvironment [58,59,60]. This is reminiscent of a recent study proposing FABP7 as a potential biomarker for predicting the prognosis and efficacy of anti-angiogenic drugs for the treatment of glioma [50]. While the underlying mechanisms warrant further investigation, it is tempting to speculate that the differential profiles of tumor immune infiltration modulated by FABP7 could contribute to the disparate outcomes observed between LGG and GBM patients. 

While high FABP7 expression has been linked to poor prognosis in glioma cancers in the existing literature, our data suggest that its role varies between LGG and GBM, reflecting distinct prognostic implications and immune interactions due to the generally different molecular and clinical characteristics of these tumor types [61,62]. However, a major limitation of this study is its reliance on correlational data derived from transcriptomic analyses and pan-cancer datasets. This approach provides valuable insights but is inherently limited in establishing causal relationships or capturing the complex, dynamic interactions of FABP7 within the tumor microenvironment. To validate and extend our findings, future studies are needed that employ functional in vivo models, including humanized mouse models using tumor cells from both GBM and LGG patients [63,64,65,66]. Such models could enable a more nuanced exploration of FABP7’s role in brain tumor progression, including its influence on immune cell infiltration and tumor–stroma interactions. Addressing these limitations through further research may clarify the therapeutic potential of targeting FABP7 in a tumor-specific manner, especially given the growing interest in developing novel FABP7 inhibitors [67] and advancing cancer immunotherapy [68,69].

## 4. Materials and Methods

### 4.1. Animals

All animal procedures were carried out in accordance with the National Institutes of Health Guide for the Care and Use of Laboratory Animals, as well as the ARRIVE and OLAW guidelines, and were approved by the WSU Institutional Animal Care and Use Committee (IACUC; ASAF# 6509). Fourteen-week-old Fabp7-KO and WT mice (both in a C57BL/6NJ background) were housed at 22 ± 2 °C with a 12:12 h light-dark cycle. They were acclimated to this light cycle for at least 10 days prior to tissue collection, with water and chow ad libitum. At ZT20, eight hours after lights-out, wild-type (WT) (*n* = 5) and *Fabp7*-knockout (Fabp7 KO) mice (*n* = 4) were euthanized, and cortical brain tissues were collected. Total RNA was prepared from these tissues and processed by the WSU Genomics Core.

### 4.2. RNA Sequencing and Data Analysis

Total RNA was purified using an RNeasy Mini Kit (Qiagen, Hilden, Germany). The integrity of the total RNA was assessed using a Fragment Analyzer (Advanced Analytical Technologies, Ankeny, IA, USA) with the High Sensitivity RNA Analysis Kit. An RNA Quality Number (RQN) from 1 to 10 was assigned to each sample to indicate its integrity or quality, with “10” indicating a perfect sample without any degradation and “1” indicating a completely degraded sample. RNA samples with RQNs ranging from 8 to 10 were used for RNA library preparation with the TruSeq Stranded mRNA Library Prep Kit (Illumina, San Diego, CA, USA). Briefly, mRNA was isolated from 1–2.5 µg of total RNA using poly-T oligos attached to magnetic beads and subjected to fragmentation, followed by cDNA synthesis, dA-tailing, adaptor ligation, and PCR enrichment. The sizes of the RNA libraries were assessed with a Fragment Analyzer using the High Sensitivity NGS Fragment Analysis Kit (Advanced Analytical Technologies, Ames, IA, USA). The concentrations of the RNA libraries were measured using the StepOnePlus Real-Time PCR System (ThermoFisher Scientific, San Jose, CA, USA) with the KAPA Library Quantification Kit (Kapabiosystems, Wilmington, MA, USA). The libraries were then diluted with RSB (10 mM Tris-HCl, pH 8.5), denatured with 0.1 N NaOH, and sequenced on a NovaSeq 6000 (Illumina, San Diego, CA, USA). The DNA was sequenced from both ends (paired-end) with a read length of 150 bp. The raw bcl files were converted to FASTQ files using the software program bcl2fastq v2.20.01, and adaptors were trimmed during the conversion. On average, 40 million reads were generated for each sample. Sequencing data (FASTQ files) were processed by trimming low-quality reads ≤ Q30, (about 3%), using Trimmomatic (version 0.39) [70] and removing rRNA sequences using SortMeRNA (version 4.3.5) [71]. The remaining reads were aligned to the Mus musculus reference genome (mm10, UCSC) using HISAT2 (version 2.2.1) [72]. Gene expression quantification and differential expression were analyzed using featureCounts (part of the Subread package, version 2.0.3) [73] and DESeq2 (version 1.36.0) [74], respectively. Further detailed methodology and RNA-seq data from our study are available from NCBI GEO (Accession #GSE271985). For analyzing transcriptomic changes induced by FABP7 overexpression in human iPSC-derived astrocyte cultures, we utilized RNA-seq data available in NCBI’s Gene Expression Omnibus (GEO; accession number GSE214628). Differential gene expression (DGEn) and differential gene expression (DGEx) analyses were conducted following established methods published in recent literature. Gene expression levels were compared between control samples and those overexpressing FABP7 using DESeq2, with the Wald test employed to generate *p*-values and Log2 fold-changes (FCs). Genes meeting the criteria of an adjusted *p*-value (padj) < 0.05 and an absolute log2FC > 1 were considered significantly regulated [10]. 

### 4.3. Gene Ontology and Pathway Enrichment Analysis

Gene Ontology (GO) and Kyoto Encyclopedia of Genes and Genomes (KEGG) pathway enrichment analyses were conducted using ShinyGO, a graphical gene set enrichment tool for animals [75]. ShinyGO facilitated the generation of the lollipop charts, hierarchical clustering trees, and pathway/network representations, illustrating comparative GO and KEGG pathway enrichment profiles for all genes significantly up- and downregulated, or genes up- and downregulated by at least 2-fold, in WT vs. *Fabp7*-KO mouse cortical tissues and control (CTL) vs. FABP7-overexpressing (FABP7 OV) human astrocytes, as indicated in the Figures.

### 4.4. Differential Gene Expression Analysis 

Using Science and Research online plot (SR plot) software (https://www.bioinformatics.com.cn/en, accessed on 1 October 2023) [76], heatmap, volcano plot, and Venn diagram representations were generated to illustrate differential gene expression profiles for genes that were significantly up- or downregulated or genes that were up- or downregulated by at least 2-fold, in WT vs. *Fabp7*-KO mouse cortical tissues and control (CTL) vs. FABP7 OV human astrocytes, as indicated in the Figures.

### 4.5. Differential Gene Expression Analysis in Normal and Cancer Tissues 

Differential expression of *FABP7* and its modulated genes (*ENO1*, *MUC1*, *COL5A1*, and *IL11*) between normal and cancer tissues were evaluated using the Gene_DE module in the TIMER (Tumor Immune Estimation Resource) database (https://cistrome.shinyapps.io/timer/, accessed on 1 October 2023) [77], an integrated web database server capable of accurately quantifying tumor purity. The Gene_DE module allows for study of the differential expression of any gene of interest between tumor (all TCGA tumors) and adjacent normal tissues. The distributions of gene expression levels are displayed using box plots. The statistical significance was computed using the Wilcoxon test and is annotated based on the number of asterisks (* *p* < 0.05; ** *p* < 0.01; *** *p* < 0.001). Genes that are upregulated or downregulated in tumors compared to normal tissues for each cancer type are identified, as displayed in gray columns, when normal data are available.

### 4.6. Assessment of the Clinical Relevance of Gene Expression Across Diverse Cancer Types

The clinical relevance of the expression of *FABP7* and its modulated genes (*ENO1*, *MUC1*, *COL5A1*, and *IL11*) across various cancer types was assessed using the Gene_Outcome module in TIMER. In addition to the Outcome module, for survival prognosis analysis, the Gene_Surv module was employed to depict survival curves for the target genes using the Cox proportional hazard model. Kaplan–Meier (KM) curve parameters were applied to evaluate the outcome significance of gene expression in brain cancers, specifically low-grade glioma (LGG) and glioblastoma (GBM). To separate the cohorts with high and low expression, the cutoff-high and cutoff-low values (50%) were employed as the expression cutoffs. Hazard ratios and *p*-values for the Cox model and log-rank *p*-values for the KM curves are shown on the KM curve plots. The analysis was adjusted for clinical factors, such as age, stage, and purity. SR plot software was used to plot the bar graphs provided from the Gene_Outcome module in the TIMER database regarding the clinical relevance of the SD-induced gene expression.

### 4.7. Analysis of the Association Between Gene Expression and Immune Infiltration

The abundance of tumor-infiltrating immune cells (TIICs) across diverse cancer types was assessed using Gene_module in the TIMER2.0 database (http://timer.cistrome.org/, accessed on 1 October 2023), developed by Dr. Xiaole Shirley Liu Lab at the Dana-Farber Cancer Institute, Boston, MA, USA). Using this module, we explored the association between the expression levels of *FABP7* and its modulated genes (*ENO1*, *MUC1*, *COL5A1*, and *IL11*) and the abundance of TIICs, including CD4+ T cells, CD8+ T cells, macrophages, Tregs, cancer-associated fibroblasts (CAFs), and myeloid-derived suppressor cells (MDSCs). Various estimation algorithms, such as Cell-type Identification by Estimating Relative Subsets of RNA Transcripts (CIBERSORT), Estimating the Proportions of Immune and Cancer cells (EPIC), Microenvironment Cell Populations-counter (MCPCOUNTER), Tumor Immune Dysfunction and Exclusion (TIDE), and Quantification of Immune Cell Types Sequencing (QUANTISEQ), were employed for the association analysis between SD-induced gene expression and immunity. The purity-adjusted Spearman’s rank correlation test was utilized to ascertain both the *p*-values and partial correlation (cor) values. Each TIIC displayed Kaplan–Meier (KM) curves for the corresponding immune infiltrates and cancer types. 

### 4.8. Profiling FABP7 Expression in Human Cancer Tissues and Cells 

For expression profiling analysis of FABP7 in various human cancer tissues and cells, we utilized The Human Protein Atlas (HPA) database (www.proteinatlas.org, accessed on 1 October 2023), which is dedicated to mapping all human proteins in cells, tissues, and organs. We utilized the Tissue Atlas module to display the expression of FABP7 protein via immunohistochemistry staining. Additionally, the Subcellular Atlas module was used to illustrate the subcellular localization of FABP7 expression.

### 4.9. Examination of FABP7 Expression at the Single-Cell Level 

For single-cell gene expression profiling analysis of *FABP7*, we utilized the Tumor Immune Single-cell Hub (TISCH) (http://tisch.comp-genomics.org/, accessed on 1 November 2023), a comprehensive web resource that enables interactive visualization of single-cell transcriptomes in the tumor microenvironment [78]. Within the “dataset” module, we visualized the expression of *FABP7* at the single-cell level in the Glioma_GSE138794 and Glioma_GSE163108 datasets.

### 4.10. Statistical Analysis

The correlation analysis was evaluated in the TIMER database using Spearman’s correlation analysis. The correlations between *FABP7/ENO1/MUC1/COL5A1/IL11* expression and abundance scores of immune cells were evaluated based on Spearman’s correlation.

## 5. Conclusions

Our study offers a detailed understanding of how FABP7 contributes to tumor progression by reshaping the oncogenic gene expression landscape within glioma cells, specifically through its influence on key cancer-associated pathways. By driving the expression of onco-immune modulatory genes, such as ENO1, MUC1, COL5A1, and IL11, FABP7 enhances tumor-associated immune suppression, particularly by facilitating the infiltration of immunosuppressive cell populations within TIME. This effect is more pronounced in low-grade glioma (LGG), where the expression of FABP7 and its regulated genes strongly correlates with poor clinical outcomes and prognosis (Table 2). Overall, our data reveal that FABP7 plays an important role not only in tumor-intrinsic metabolic and oncogenic processes but also in shaping the tumor’s immune landscape, suggesting that targeting FABP7 could provide therapeutic strategies for modulating both tumor growth and immune evasion in brain cancers.

## Figures and Tables

**Table 1:** FABP7-regulated onco-driver genes and their roles in brain cancer.

Gene	Function	Role in Brain Tumors (Glioma/GBM)	Refs.
*ENO1*	Glycolysis	Promotes tumor growth and metastasis	[37,38]
*MUC1*	Cell adhesion, signaling	Enhances tumor progression and drug resistance	[39,40]
*COL5A1*	Extracellular matrix	Promotes tumor invasion and metastasis	[41,42]
*COL11A1*	Extracellular matrix	Enhances tumor progression, metastasis, and tumor immunosurveillance	[43,44]
*IL11*	Cytokine	Promotes tumor growth, angiogenesis, and tumor immunosurveillance	[45,46]

**Table 2:** Gene correlation of FABP7 with modulated factors and their prognostic significance in LGG and GBM.

Gene	Gene Correlation with FABP7	Correlation with LGG Prognosis	Correlation with GBM Prognosis
*FABP7*	Significantly positive in LGG, but not GBM	Significantly positive	Non-significant
*ENO1*	Significantly positive in LGG, but not GBM	Significantly positive	Significantly positive
*MUC1*	Weakly positive in LGG but not GBM	Significantly positive	Non-significant
*COL5A1*	Significantly positive in LGG but not GBM	Significantly positive	Non-significant
*IL11*	Significantly positive in LGG but not GBM	Weak/Non-significant	Non-significant

## Data Availability

RNA-seq raw data were deposited in the Gene Expression Omnibus (GEO) repository (accession number GSE271985). The authors declare that all data supporting the results in this study are available within the paper and its supporting information.

## Supplementary Materials

The supporting information can be downloaded at: https://www.mdpi.com/article/10.3390/ijms252212231/s1.
