# Supplementary material for: Integrated Transcriptome Profiling and Pan-Cancer Analyses Reveal Oncogenic Networks and Tumor-Immune Modulatory Roles for FABP7 in Brain Cancers
Source: Int J Mol Sci. 2024 Nov 14;25(22):12231. doi: 10.3390/ijms252212231 (PMC11594725; doi:10.3390/ijms252212231)
Supplement: Supplementary file 1 [file ijms-25-12231-s001.zip › Supplementary Figures (IJMS, Lee et al)_revised.pdf]

Supplementary Figures

Figure S1

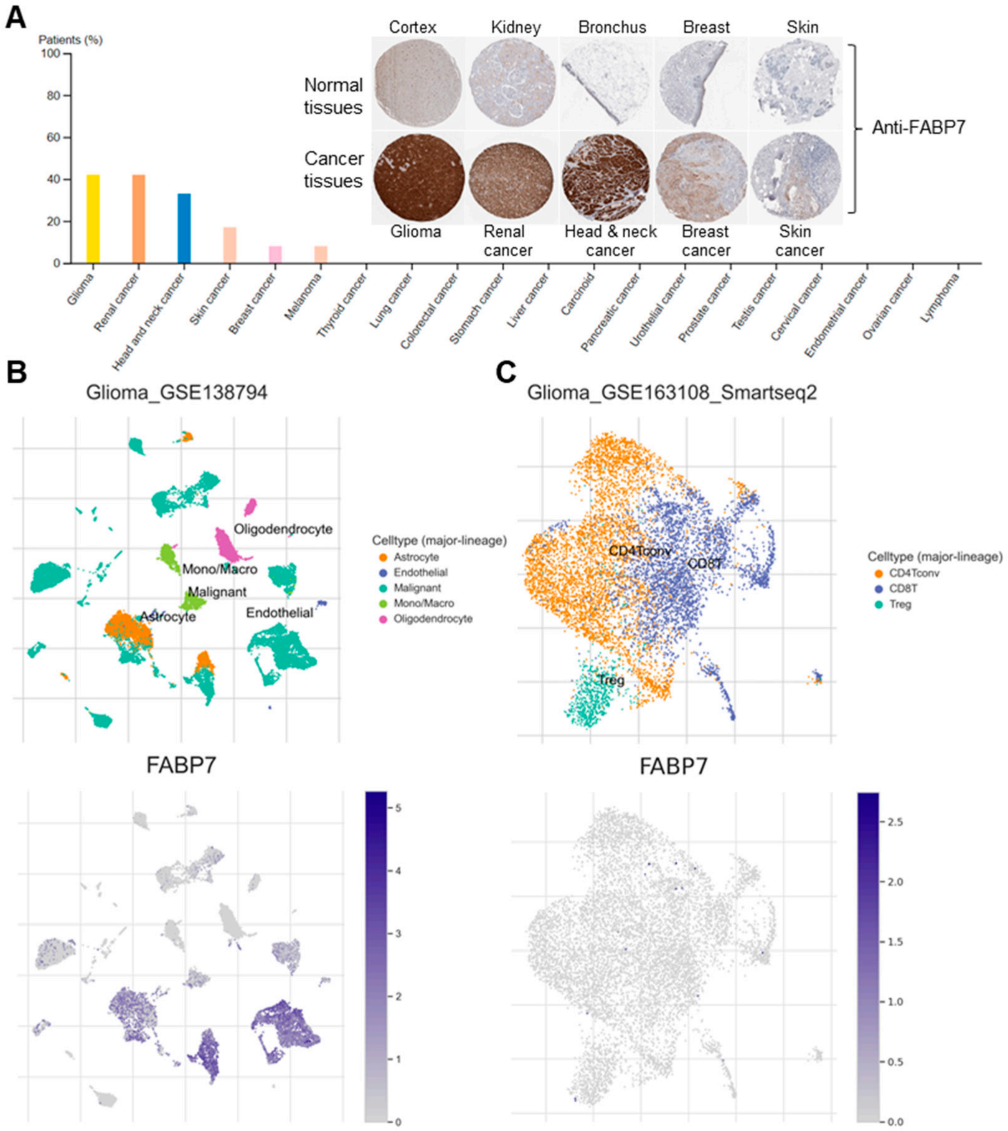

Figure S1. FABP7 is highly expressed in astrocytes and malignant gliomas. (**A**) Relative expression profiles of FABP7 across multiple patient tumors. The inset shows representative immunohistochemical staining for FABP7 in normal (upper panels) and tumor tissues (lower panels). Data are sourced from the Human Protein Atlas (https://www.proteinatlas.org). (**B, C**) FABP7 expression at the single-cell level, depicted through Uniform Manifold Approximation and Projection (UMAP) plots. These plots illustrate cellular clusters and FABP7

expression in human glioma cells derived from single-cell RNA sequencing (Gene Expression Omnibus Series datasets GSE138794 (B) and GSE163108 (C)). Data were sourced from the Tumor Immune Single-cell Hub (TISCH) (http://tisch.comp-genomics.org). Abbreviations: Mono, Monocytes; Macro, Macrophages; CD4Tconv, CD4+ T conventional cells; CD8T, CD8+ T cells; Treg, Regulatory T cells.

Figure S2

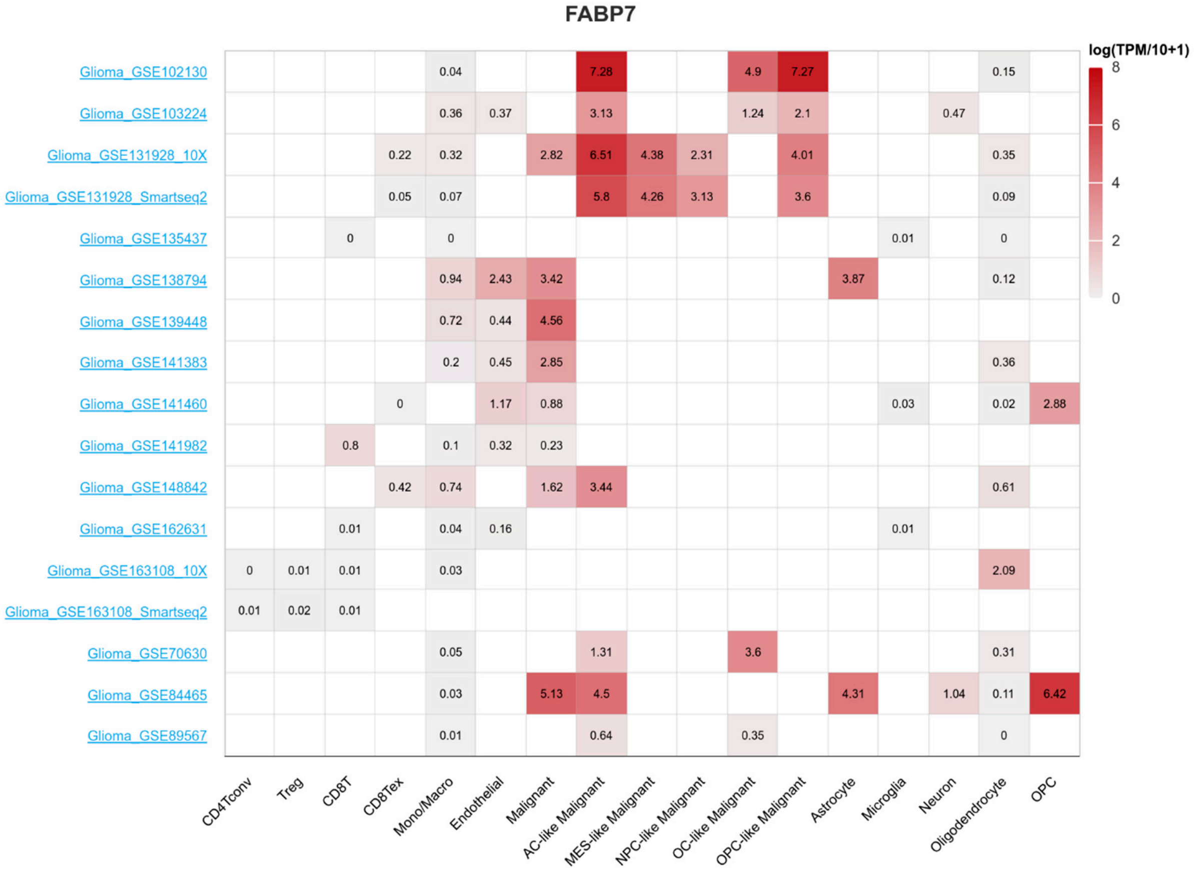

Figure S2. ***FABP7* expression at the single-cell level.** *FABP7* expression levels in various cell lineages based on single-cell RNA sequencing data from the Gene Expression Omnibus (GSE) are shown. Data was obtained using the Tumor Immune Single-Cell Hub (TISCH) (http://tisch.comp-genomics.org/).

Figure S3

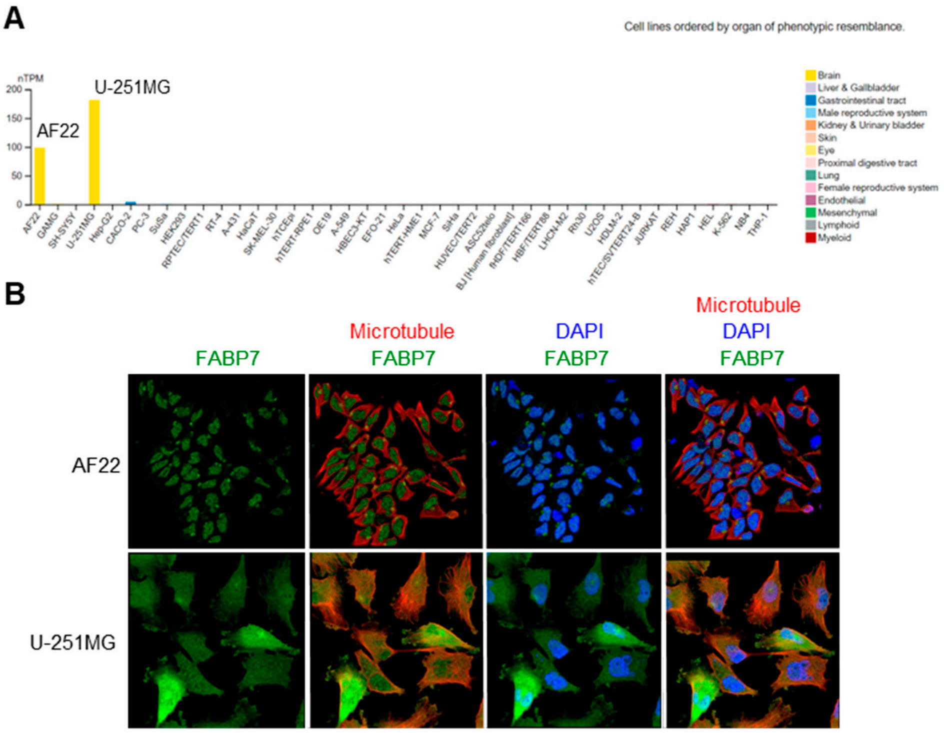

Figure S3. FABP7 is highly expressed in malignant brain tumor cells with intracellular localization that is distinct from neural stem cells. (**A**) FABP7 mRNA expression profiles of multiple established cell lines with phenotypic resemblance to various organs or tumor tissue are shown. nTPM, normalized transcripts per million. (**B**) Representative images are showing the subcellular localization of FABP7 in the AF22 neuroepithelial stem (NES) cell line derived from human in-duced pluripotent stem cells (iPSCs) and U-251MG glioblastoma cells, as determined by immunofluorescent staining. Immunofluorescent staining was performed using specific antibodies against FABP7 (green) and microtubules (red; cytoplasmic marker), along with DAPI stain (blue; nuclear marker). These data (A, B) were obtained from the Human Protein Atlas (https://www.proteinatlas.org).

Figure S4

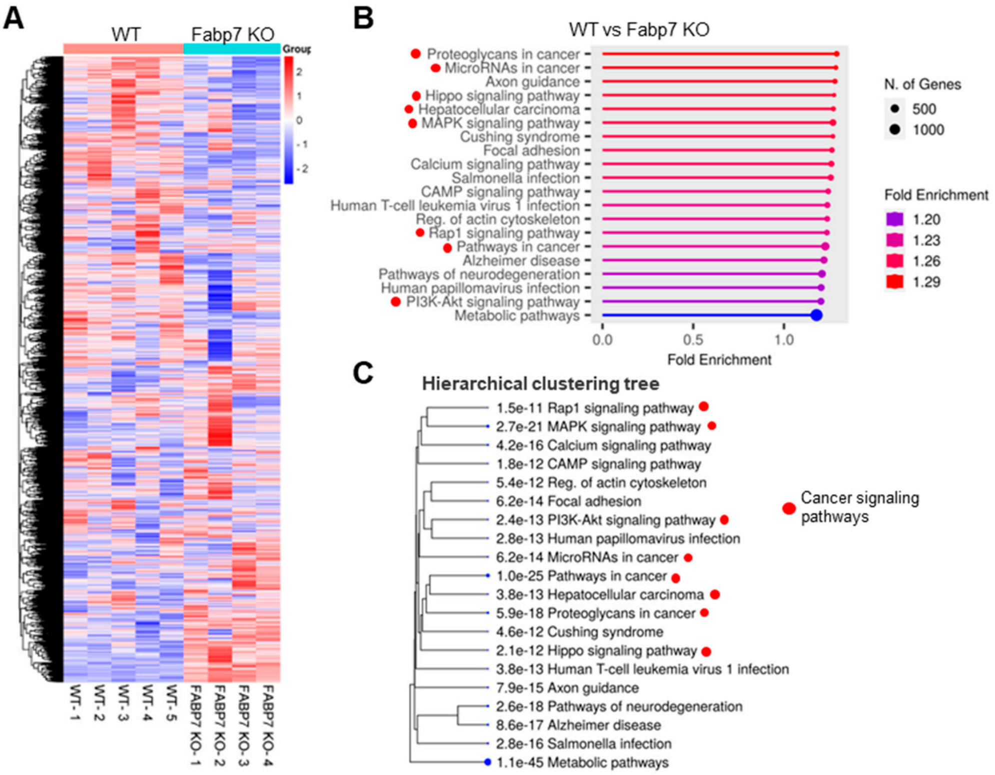

Figure S4. Ablation of *Fabp7* in mice alters gene expression profiles associated with cancer-related pathways in brain tissue. (**A**) Heatmap illustrating the differential expression profiles of detected genes in RNA sequencing analysis of cortical wildtype (WT) and *Fabp7* knockout (*Fabp7* KO) brain tissues. (**B**) Lollipop chart representing the Gene Ontology (GO) and Kyoto Encyclopedia of Genes and Genomes (KEGG) pathway enrichment profiles for all genes that were either up- or down-regulated in *Fabp7* KO compared to WT mouse cortical tissues. Significant pathways (false discovery rate (FDR) ≤ 0.05) were sorted, and the top 20 pathways (with the lowest FDR values) were further sorted based on Fold Enrichment scores. The red circles to the left of the pathway labels indicate cancer-associated pathways. (**C**) Hierarchical clustering tree of the data shown in (B). The red rectangular box highlights cancer-associated pathways.

Figure S5

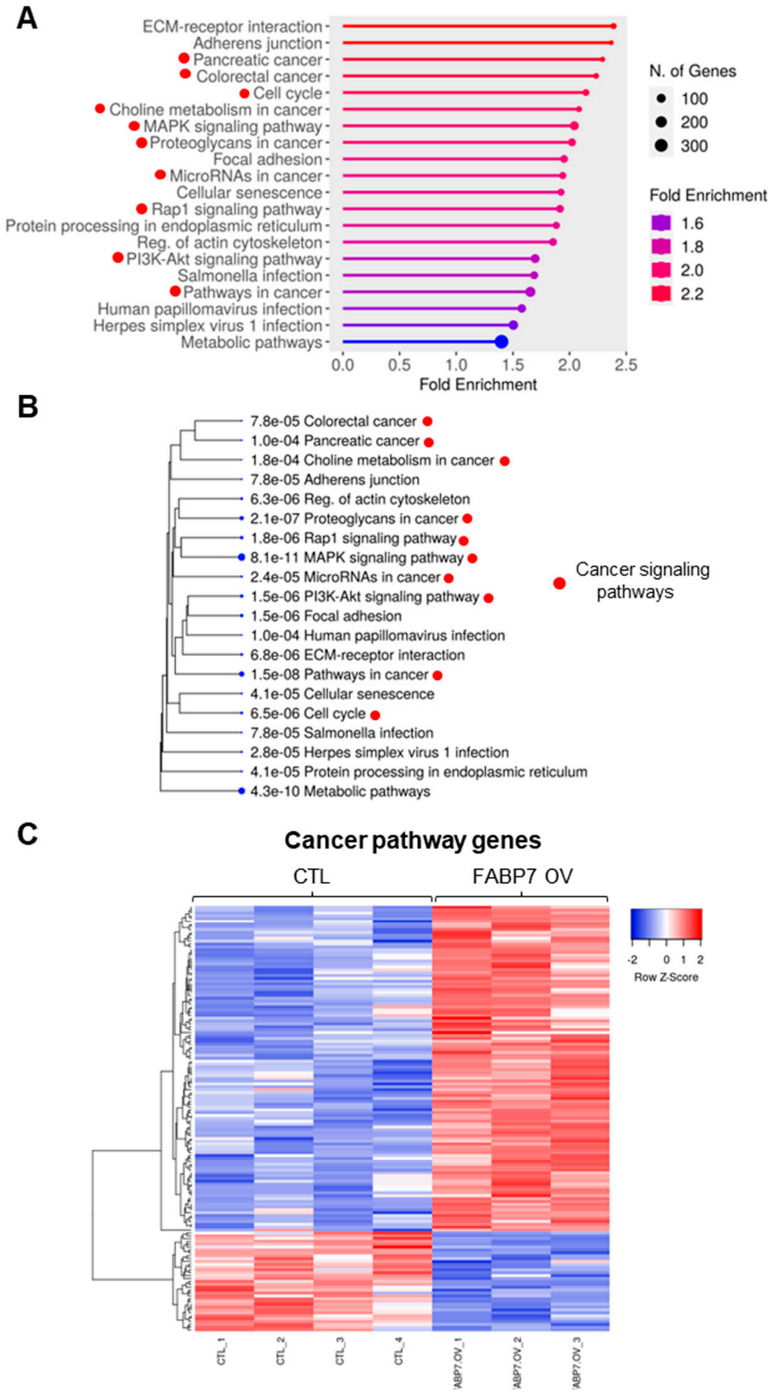

Figure S5. FABP7 overexpression upregulates multiple genes associated with cancer signaling pathways in human astrocytes. (**A**) Lollipop chart representing the Gene Ontology

(GO) and Kyoto Encyclopedia of Genes and Genomes (KEGG) pathway enrichment profiles for genes that were significantly up- or downregulated in FABP7-overexpressing (FABP7 OV) human astrocytes compared to control (CTL) astrocytes. Significant pathways (false discovery rate (FDR) ≤ 0.05) were sorted, and the top 20 pathways (with the lowest FDR values) were further sorted based on Fold Enrichment scores. Red circles indicate cancer-associated pathways. (**B**) Hierarchical clustering trees representing the pathway enrichment profiles in (A). (**C**) Heatmap illustrating the differential gene expression in CTL (*n*=4) vs FABP7 OV (*n*=3) human astrocytes for the enriched genes associated with cancer signaling pathways, as indicated by the red circles in (A) and (B).

Figure S6

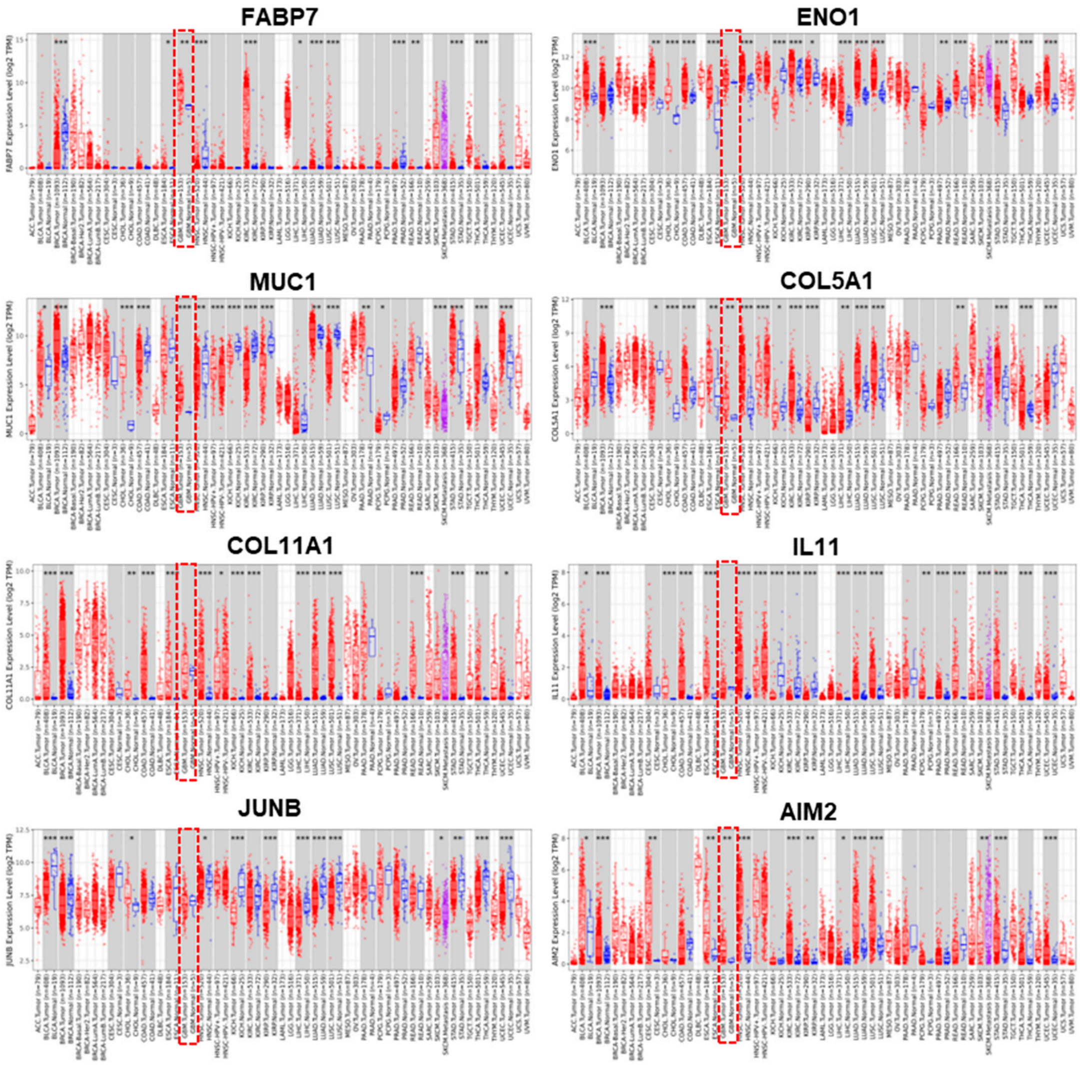

Figure S6. Expression of *FABP7* and its modulated factors in tumors and their adjacent normal tissues across all tumor types in The Cancer Genome Atlas (TCGA). The expressions of *FABP7* and its modulated genes, as indicated, in normal (blue) and cancer tissues (red) from TCGA tumors were determined using the Gene_DE module in the TIMER (Tumor Immune Estimation Resource) database (https://cistrome.shinyapps.io/timer/). The indicated genes show notably elevated expression levels in various cancers compared to adjacent normal tissues. The expression profiles of genes in glioblastoma (GBM) tumor and adjacent normal tissues are boxed with a dashed red line. The distributions of gene expression levels are displayed using

box plots. Statistical significance was computed using the Wilcoxon test and is indicated with asterisks: *$p < 0.05$; **$p < 0.01$; ***$p < 0.001$. Abbreviations: ENO1, Enolase 1; MUC1, Mucin 1; COL5A1, Collagen Type V Alpha 1 Chain; COL11A1, Collagen Type XI Alpha 1 Chain; IL11, Interleukin 11; AIM2, Absent in Melanoma 2; JUNB, Jun B Proto-Oncogene.

Figure S7

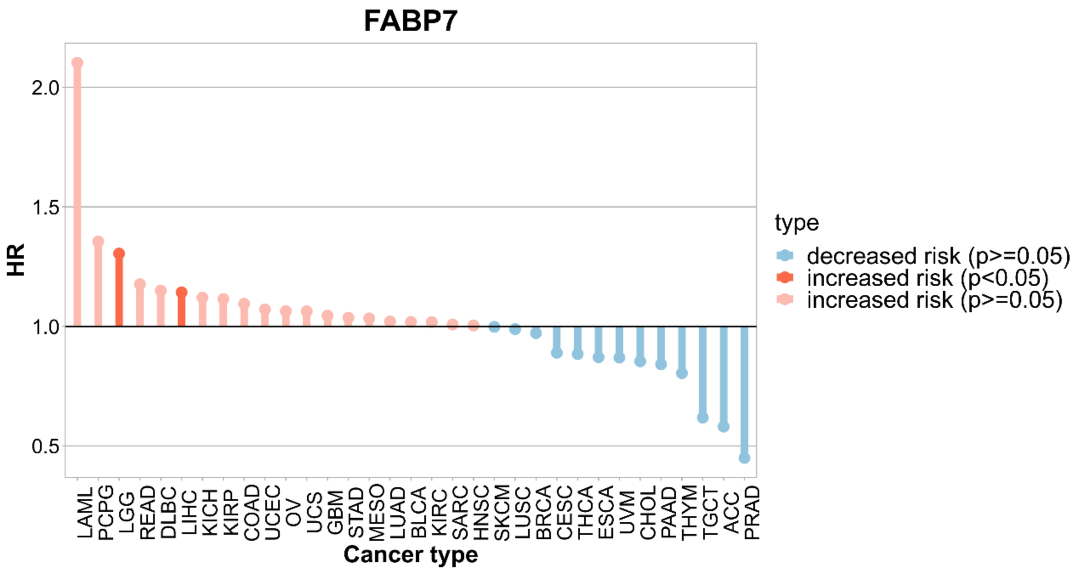

Figure S7. Expression of *FABP7* shows a significantly higher correlation with increased risk in low-grade glioma (LGG) compared to other cancer types. Lollipop graph depicting the association between *FABP7* expression and elevated cancer risk by measuring the hazard ratio (HR) using survival analysis. This analysis compares the risk of events such as death or cancer recurrence across various cancer types, as indicated, based on data from The Cancer Genome Atlas (TCGA) database. Significantly increased risk ($p < 0.05$) is represented by dark brown coloration for low-grade glioma (LGG) and liver hepatocellular carcinoma (LIHC).

Figure S8

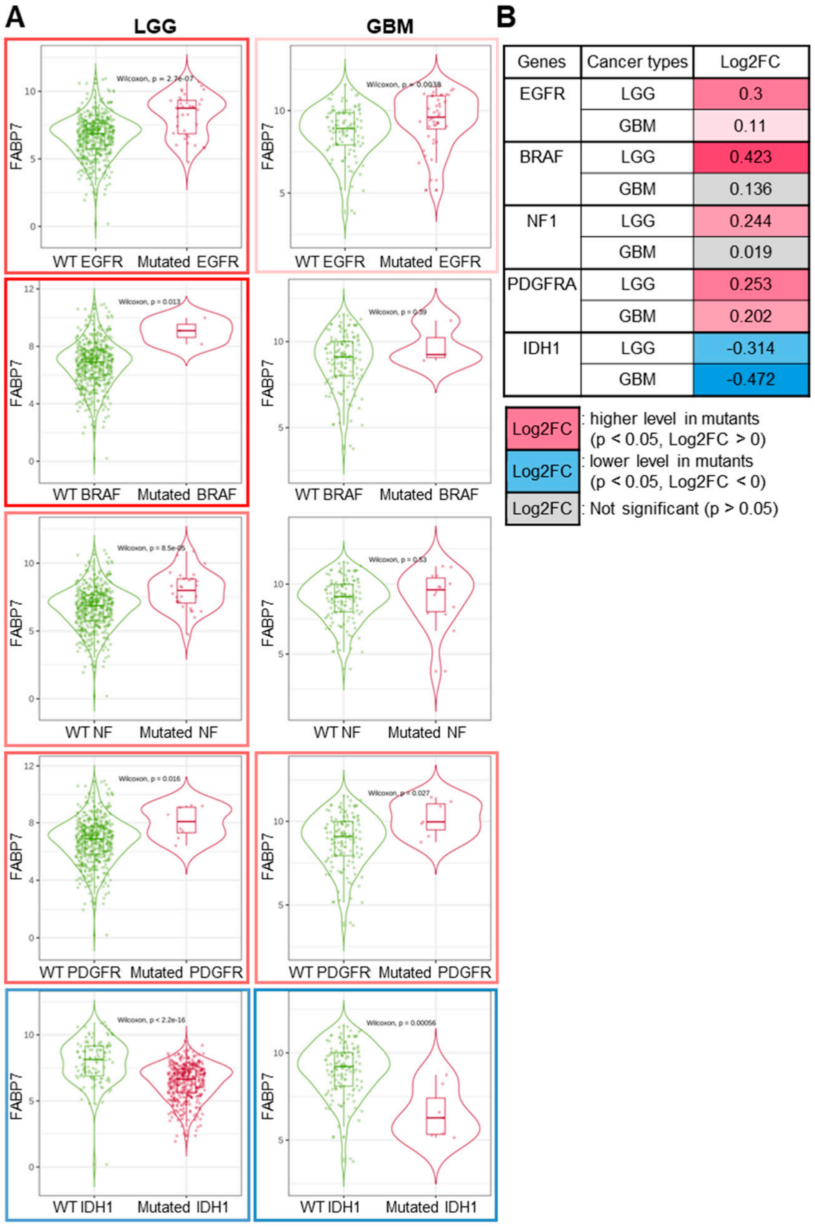

B

Genes	Cancer types	Log2FC
EGFR	LGG	0.3
	GBM	0.11
BRAF	LGG	0.423
	GBM	0.136
NF1	LGG	0.244
	GBM	0.019
PDGFRA	LGG	0.253
	GBM	0.202
IDH1	LGG	-0.314
	GBM	-0.472

Log2FC	: higher level in mutants ($p < 0.05$, Log2FC > 0)
Log2FC	: lower level in mutants ($p < 0.05$, Log2FC < 0)
Log2FC	: Not significant ($p > 0.05$)

Figure S8. FABP7 expression shows a stronger correlation with tumor mutation burden in LGG compared to GBM. (**A**) Boxplots showing the gene expression profiles of FABP7 in LGG (n=511) and GBM (n=148) tumors containing wildtype (WT) or mutated versions of the indicated proteins that are well-known to cause these brain cancer types. These profiles were obtained using the Gene_Mutation module in the TIMER database. Positive and negative correlations ($p < 0.05$), based on Wilcoxon signed-rank tests, are indicated by boxes of varying

shades of red and blue, respectively, to indicate significance, as shown in (B). (**B**) Table of the Log2 fold change (FC) values indicating the varying degrees of association between FABP7 expression and mutations in the indicated genes in LGG and GBM, as described in (A) (see Materials and Methods for details). Abbreviations: EGFR, Epidermal Growth Factor Receptor; BRAF, B-Raf Proto-Oncogene, Ser-ine/Threonine Kinase; NF1, Neurofibromin 1; PDGFRA, Platelet-Derived Growth Factor Receptor Alpha; IDH1, Isocitrate Dehydrogenase (NADP(+)) 1.
